# Relation between the risk factors for the severity of denture stomatitis and quality of life of complete edentulous individuals: a cross-sectional study

**DOI:** 10.1590/1678-7757-2023-0192

**Published:** 2023-12-18

**Authors:** Eleonora Nardi CAMPOS, Lorena Mosconi CLEMENTE, Pillar Gonçalves PIZZIOLO, Viviane de Cássia OLIVEIRA, Ana Paula MACEDO, Evandro WATANABE, Cláudia Helena SILVA-LOVATO, Adriana Barbosa RIBEIRO

**Affiliations:** 1 Universidade de São Paulo Faculdade de Odontologia de Ribeirão Preto Departamento de Materiais Dentários e Prótese Ribeirão Preto São Paulo Brasil Universidade de São Paulo. Faculdade de Odontologia de Ribeirão Preto. Departamento de Materiais Dentários e Prótese. Ribeirão Preto, São Paulo.; 2 Universidade de São Paulo Faculdade de Odontologia de Ribeirão Preto Departamento de Dentística Restauradora Ribeirão Preto São Paulo Brasil Universidade de São Paulo. Faculdade de Odontologia de Ribeirão Preto. Departamento de Dentística Restauradora. Ribeirão Preto, São Paulo.

**Keywords:** Denture Stomatitis, Risk factors, Age, Complete dentures, Quality of life

## Abstract

**Objective:**

To assess the association between risk factors for developing denture stomatitis (DS) and oral health-related quality of life (OHRQoL) in complete denture wearers.

**Methodology:**

Participants of both sexes, wearing complete dentures, were classified using the modified Newton classification for the absence or the severity of DS and allocated to groups Normal or zero, IA, IB, II, and III. Lifestyle, oral and denture history, and medication use were assessed using specific questionnaires; clinical parameters such as anatomical characteristics of support were evaluated with the Kapur classification; salivary flow (SF) was calculated by the volume of unstimulated saliva per minute; and microbial load was determined by counting colony forming units (CFU) of target microorganisms present in the biofilm collected from dentures and palate. OHIP-EDENT assessed the OHRQoL. Kendall's tau_b and Spearman tests were applied with a significance level of 5%.

**Results:**

184 patients (143 female and 41 male) aged 65.5 ± 6.8 years were evaluated. Positive correlations were found for sex (women; p=0.013, r=0.16), individuals who started to consume alcoholic beverages as a young adult (18–27 years) (p=0.008, r=0.22), CFU of *Candida* spp. (p<0.001, r=0.27 denture; p<0.001, r=0.31 palate); *Candida albicans* (p=0.004, r=0.22 denture; p=0.003, r=0.25 palate), and *Candida glabrata* (p=0.004, r=0.22 denture; p=0.001, r=0.27 palate). Moreover, negative correlations with DS were found for CFU of *Staphylococcus* spp. (p=0.004, r=-0.20 palate) and enterobacteria (p=0.002, r=-0.24 palate), as well as a negative correlation between SF (p=0.009, r=-0.193) and DS. The CFU of Staphylococcus spp. and enterobacteria on the palate significantly correlated with OHRQoL.

**Conclusion:**

Being female, consuming alcoholic beverages as a young adult, CFU of *Candida* spp., *Candida albicans*, *Candida glabrata*, and salivary flow may be the most significant risk factors for DS. The microbial load of *Staphylococcus* spp. and enterobacteria seems to influence the quality of life for complete denture wearers.

## Introduction

Although treatment with complete dentures provides patients with a better quality of life,^1^ these devices may be associated with oral lesions, the most common of which are denture stomatitis (DS),^2,3^ angular cheilitis, and traumatic ulcers.^4^ DS is clinically characterized by different degrees of chronic inflammation of the tissue that supports the complete denture, usually located in the maxillary ridge and palate;^5-8^ it can be asymptomatic or manifest with burning in the palate region, especially when swallowing.^9^

The degree of DS can be diagnosed according to modified Newton’s classification, by the attribution of scores.^7^ The modified Newton’s classification introduces two subtypes for Newton type I, resulting in: type 0: healthy mucosa; type IA: petechiae in normal palatal tissue, usually found around the orifices of the ducts of the palatal mucous glands; type IB: localized area of inflammation in the denture-bearing area; type II: generalized area of inflammation in the denture-bearing area; and type III: hyperplasic palatal surface with inflammation in the denture-bearing area.^7^Among the etiological factors, local factors stand out, such as fungal infection, especially for *Candida* spp.,^2,3,10-14^ biofilm accumulation on dentures,^3,13,14^ poor denture hygiene, mucosal trauma due to adaptation problems, or the continuous and nocturnal use of dentures.^3,9^

Systemic factors that affect the immune system can be cited as the cause of DS.^11,15^ Specifically, elderly patients with diabetes have been reported to be around 4.4 times more likely to develop denture stomatitis than a healthy patient.^14^ Patients who use prescription drugs such as antidepressants, antihypertensives, diuretics, or have other conditions related to xerostomia, have a higher prevalence of stomatitis associated with dentures.^14^However, only a few factors have been statistically associated with DS, and it is essential to consider age, sex, age of dentures, and time of use to determine the possible risk factors for DS.^4,8,14,16^Most patients positive for DS do not report specific symptoms of the disease, so they often remain undiagnosed and untreated for extended periods,^17^ which can complicate their systemic health^18^ and impact the quality of life.^19^

Although the literature states that local and systemic factors are associated with the cause of DS, few studies have investigated, with clinical trials, the relationship between risk factors of DS and OHRQoL.^19^ Therefore, the primary aim of this study was to evaluate the socioeconomic profile and assess the association between risk factors such as lifestyle, oral and denture history, clinical parameters, medication use, salivary flow, and microbial load parameters of completely edentulous patients who were healthy or with different severities of DS. The secondary aim was to correlate all the variables with OHRQoL. This information may contribute to enabling more appropriate management in the prevention and treatment of DS, as well as helping to maintain these individuals’ oral and denture hygiene, especially in the promotion of alternatives aimed at public health procedures for this population composed mainly of the elderly. The null hypothesis tested was that socioeconomic profile, lifestyle, oral and denture history, clinical parameters, medication use, salivary flow, and microbial load would be similar for healthy patients and those with different severities of Denture stomatitis, and that these factors would not influence OHRQoL.

## Methodology

### Study population

This cross-sectional study was approved by the ethics committee at the School of Dentistry of Ribeirão Preto, University of São Paulo (CAAE: 3712418.1.0000.5419). All participants gave fully informed written consent and were recruited from August 2018 to February 2023.

The inclusion criteria were that the participants were of both sexes, completely edentulous, wore complete dentures, and were healthy or positive for different severities of DS, according to the modified Newton Classification.^7^ The exclusion criteria were individuals with dentures with adaptation problems, relining, repairs or fractures, and subjects with oral mucosal lesions different from DS, such as denture-induced fibrous hyperplasia, papillomas, or traumatic ulcerations associated with denture bases.

### Data collection

Two previously calibrated researchers classified the degree of DS by analysis of PowerPoint files containing photographs of the palate of participants without the identifiable name or clinical information (C.H.S-L., H.F.O.P.). Standardized photographs of the palate were taken (C.B.A., A.B.R.) using a digital camera with the focus on the median raphe region (Canon EOS, Canon EF Macro 100 mm / 2: 8 lens and Flash Canon ML3 Circular), which were then transferred to a computer.^20^The socio-demographic information, habits and lifestyle, medication reports, dental history, and information about the time of use of dentures and how individuals clean them were registered in specific questionnaires.

The sample size estimate was based on a previous study^20^ that calculated colony-forming unit (CFU) counts following log10 transformation. The parameters showing a minimally significant difference of 1 transformed unit and a standard deviation of 2.2 led to a total sample size of at least 15 participants per group to ensure a power of 80% (a=.050; b=.200) and to reject the null hypothesis.

The ridge, the fibrous tissues, and the muscular insertions were evaluated separately during the physical examination by researchers (L.M.C., A.B.R.) according to the methodology described by *Kapur*.^21^ The maxillary and mandibular ridge was assessed on a scoring scale from 1 to 4, with 1 being the most unfavorable clinical scenario and 4 the most favorable. The supporting tissues and muscle insertions were evaluated according to a scale of 1 to 3. The clinical score for each case was obtained by adding the values attributed to the maxilla and mandible, and the result (total) was assigned to the following conditions: unfavorable/bad clinical conditions (less than 14); satisfactory clinical conditions (between 14 and 17); and correct clinical conditions (higher than 17).^21^All saliva samples were collected from 9:00 to 11:00 AM. The participants were asked to spit into a tube for 10 minutes to collect unstimulated total saliva. The volume of each patient’s saliva was recorded to evaluate the salivary flow rate in mL/minute.

The denture biofilm was collected by desorption method with a sterile brush (Tek, Johnson & Johnson do Brasil Indústria e Comércio de Produtos para Saúde Ltda., SJ dos Campos, SP, Brazil) and buffer solution (PBS-phosphate buffered saline) in an aseptic zone.^22^ To collect the palate samples, a sterile cytology brush was rubbed in the palatal regions affected by DS for 1 minute.^7^ Then, the active tip was removed and stored in a sterile tube containing 5 mL of PBS solution. The solutions with the samples collected from the dentures and the palate were stirred for 1 minute with the aid of a Vortex shaker (Phoenix® - AP56, Ind. E Com. De Equip. Científicos Ltda, Araraquara, SP, Brazil). Next, 50 μL of the solutions were diluted in 450 μL of PBS, obtaining serial dilutions of 10^-1^ to 10^- 3^ and added to Petri dishes with the following specific culture media: CHROMagar (CHROMagar™ *Candida*, Becton Dickinson, Paris, França) for isolation of *Candida* spp.; MacConkey Agar (Himedia Laboratories PVI Ltd, Mumbai, Índia) for isolation of Gram-negative microorganism; Manitol Agar Salt (Kasvi Imp. e Dist. de Prod. para Laboratórios Ltda, Curitiba, Brasil) for isolation of *Staphylococcus* spp.; and SB20 Agar Modified by casitone for isolation of *Streptococcus mutans*. The plates were incubated in a microbiological incubator (De Leo Equipamentos Laboratoriais, Porto Alegre, RS, Brazil) at 37ºC for 48 hours, and *S. mutans* was placed in a microaerophilic environment in an anaerobic jar (Permution, Curitiba, PR, Brazil). Microorganisms were identified by evaluating the macroscopic morphology of the colonies in differential selective culture media.

OHRQoL was evaluated by the Brazilian version of the Oral Health Impact Profile in Edentulous Adults by OHIP-EDENT inventory.^23^ This instrument comprises 19 items that can be answered on a 3-point Likert scale and provides a summary score ranging from 0 to 38. Lower scores represent a better OHRQoL, which has been conceptually defined as the low impact of oral conditions on oral functions and well-being. Items in the translated version allowed for grouping into 4 subscales, representing different domains or dimensions of the perceived impact, such as masticatory-related complaints or psychological disability and discomfort.

### Data analysis

The data showed a non-normal distribution (Shapiro-Wilks test). Kendall’s tau_b test was selected to correlate sociodemographic characteristics, lifestyle, oral and denture history, *Kapur* index, and salivary flow with DS, considering the degree of inflammation. Spearman’s test was used for correlated microbial load with the degree of DS and all factors with OHRQoL. Statistical analyses were performed using statistical analysis software (SPSS Statistics version 21. IBM Corp), considering α<0.05.

## Results

**Figure 1:**
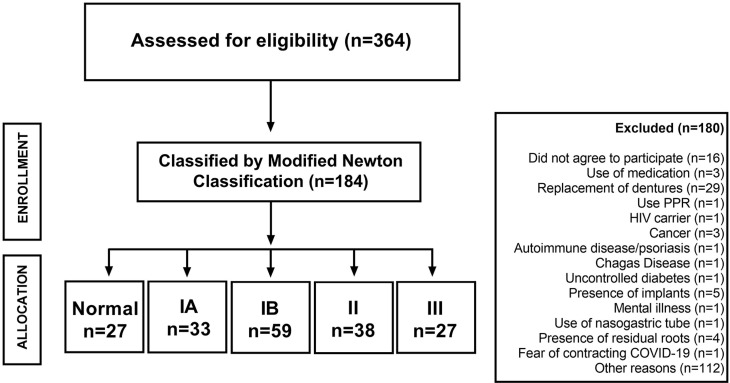
Flowchart showing the enrollment of participants 0, IA, IB, II, III are degrees of DRS according to the Newton Modified Classification. Being, 0: healthy mucosa; IA: petechiae in normal palatal tissue; IB: localized area of inflammation of the denture-bearing area; II: generalized area of inflammation of the denture-bearing area; III: hyperplasic palatal surface with inflammations of the denture-bearing area.

The final sample consisted of 184 participants (Normal: n=27; IA: n=33; IB: n=59; II: n=38; III: n=27), as shown in Figure 1, and response variables were collected from all participants. The mean age was 65.5 ± 6.8 years, being, in general, women (n=143, mean age of 66.4 ± 7,1) who showed a higher degree of DS (r=0.166; p=0.013) than men (n=41, mean age of 66.4 ± 5.4). The factors age (r=-0.029, p=0.637), marital status (r=0.063, p=0.302), level of education (r=-0.009, p=0.885), and income (r=-0.095; p=0.152) were not significantly correlated with degree of DS (Table 1). No difference was observed in the distribution of patients for the factor of “lives with” and DS (r=0.011, p=0.868).

**Table 1:** Sociodemographic data of the sample expressed in absolute numbers and correlation to DS

	Frequency n (%) of DS' degree					Correlation to DS	
Variable	0	IA	IB	II	III	r	P-value
**Gender**						0.166	0.013*
Female	18 (66.7)	23 (69.7)	47 (79.7)	29 (76.3)	26 (96.3)		
Male	9 (33.3)	10 (30.3)	12 (20.3)	9 (23.7)	1 (3.7)		
**Age**						-0.029	0.637
up to 59 years	6 (22.2)	5 (15.2)	8 (13.6)	6 (15.8)	3 (11.1)		
60-64 years	4 (14.8)	7 (21.2)	11 (18.6)	7 (18.4)	8 (29.6)		
65-69 years	6 (22.2)	12 (36.4)	18 (30.5)	12 (31.6)	11 (40.7)		
>70 years	11 (40.7)	9 (27.3)	22 (37.3)	13 (34.2)	5 (18.5)		
**Marital Status**						0.063	0.302
Single	2 (7.4)	5 (15.2)	5 (8.5)	4 (10.5)	3 (11.1)		
Married	17 (63.0)	19 (57.6)	25 (42.4)	18 (47.4)	15 (55.6)		
Divorced	5 (16.1)	3 (9.1)	14 (23.7)	7 (18.4)	2 (7.4)		
Separated	2 (7.4)	2 (6.1)	3 (5.1)	2 (5.3)	1 (3.7)		
Widowed	1 (3.7)	3 (9.1)	12 (20.3)	7 (18.4)	6 (22.2)		
Others	0 (0.0)	1 (3.0)	0 (0.0)	0 (0.0)	0 (0.0)		
Lives With						0.011	0.868
Alone	3 (11.1)	5 (15.2)	15 (25.4)	5 (13.2)	2 (7.4)		
Friend	1 (3.7)	1 (3.0)	2 (3.4)	0 (0.0)	2 (7.4)		
Family	23 (85.2)	27 (81.8)	42 (71.2)	33 (86.8)	23 (85.2)		
**Education**						-0.009	0.885
Illiterate	3 (11.1)	5 (15.2)	5 (8.5)	1 (2.6)	4 (14.8)		
Preschool	1 (3.7)	9 (27.3)	20 (33.9)	11 (28.9)	5 (18.5)		
Elementary and Middle school	18 (66.7)	16 (48.5)	28 (47.5)	16 (42.1)	15 (55.6)		
High school	2 (7.4)	1 (3.0)	5 (8.5)	8 (21.1)	3 (11.1)		
Higher education	3 (11.1)	2 (6.1)	1 (1.7)	2 (5.3)	0 (0.0)		
**Income**						-0.095	0.152
1 to 3 salaries	25 (92.6)	29 (87.9)	57 (96.6)	37 (97.4)	26 (96.3)		
4 to 7 salaries	2 (7.4)	4 (12.1)	2 (3.4)	0 (0.0)	1 (3.7)		
7 to 10 salaries	0 (0.0)	0 (0.0)	0 (0.0)	1 (2.6)	0 (0.0)		

Kendall's tau_b test (p<0.05)*. DS: Denture Stomatitis. 0: healthy mucosa; IA: petechiae in normal palatal tissue; IB: localized area of inflammation of the denture-bearing area; II: generalized area of inflammation of the denture-bearing area; III: hyperplasic palatal surface with inflammations of the denture-bearing area.

As for habits and lifestyle, negative correlations were identified with alcoholic drink consumption (r=-0.149; p= 0.006), and patients that never and rarely partake in alcoholic beverages presented higher degrees of DS. However, among patients who consumed alcoholic drinks, patients who started drinking alcohol when they were young adults (18–27 years) had a higher degree of DS (r=0.022, p=0.008) (Table 2). The medication frequency reports (Table 3) show that only patients who are users of antidepressants showed a negative correlation with the salivary flow rate (r=-0.129; p=0.038), and SF presented a significantly negative relation with DS (r=-0.194, p=0.009).

**Table 2:** Correlation of lifestyle and degree of DS

Characteristic	Frequency n (%) of DS' degree					Correlation to DS	
Lifestyle (LS)						r	p-value
	**0**	**IA**	**IB**	**II**	**III**		
**LS 1.**						0.009	0.887
No	11 (40.7)	13 (39.4)	25 (42.4)	14 (36.8)	11 (40.7)		
Yes	16 (59.3)	20 (60.6)	34 (57.6)	24 (63.2)	16 (59.3)		
**LS 2.**						-0.017	0.837
less than 18 years	13 (76.5)	14 (63.6)	23 (65.7)	17 (68.0)	13 (81.2)		
18 to 27 years	4 (23.5)	5 (22.7)	9 (25.7)	6 (24.0)	1 (6.2)		
28 to 37 years	0 (0.0)	3 (8.6)	3 (8.6)	2 (8.0)	1 (6.2)		
Above 37 years	0 (0.0)	0 (0.0)	0 (0.0)	0 (0.0)	1 (6.2)		
**LS 3.**						0.123	0.116
Up to 15 years	0 (0.0)	6 (27.3)	3 (8.8)	1 (4.0)	3 (18.8)		
16 to 30 years	6 (35.3)	6 (27.3)	5 (14.7)	1 (4.0)	1 (6.2)		
Over 30 years	5 (29.4)	5 (22.7)	12 (35.3)	15 (60.0)	5 (31.2)		
Smoker	6 (35.3)	5 (22.7)	14 (41.2)	8 (32.0)	7 (43.8)		
**LS 4.**						-0.149	0.006*
Never	8 (29.6)	18 (54.5)	34 (58.6)	21 (56.8)	16 (59.3)		
Rarely	4 (14.8)	7 (21.2)	9 (15.5)	9 (24.3)	8 (29.6)		
Occasionally	10 (37.0)	5 (15.2)	13 (22.4)	4 (10.8)	3 (11.1)		
Often	1 (3.7)	2 (6.1)	1 (1.7)	2 (5.4)	0 (0.0)		
Oftentimes	4 (14.8)	1 (3.0)	1 (1.7)	1 (2.7)	0 (0.0)		
**LS 5.**						0.222	0.008*
Under 18	10 (52.6)	7 (46.7)	8 (30.8)	3 (17.6)	2 (18.2)		
18 to 27 years	5 (26.3)	7 (46.7)	9 (34.6)	9 (52.9)	5 (45.5)		
28 to 37 years	2 (10.5)	1 (6.7)	5 (19.2)	2 (11.8)	2 (18.2)		
Above 37 years	2 (10.5)	0 (0.0)	4 (15.4)	3 (17.6)	2 (18.2)		

Kendall's tau_b test (p<0.05)*. DS: Denture Stomatitis. LS 1. Have you ever smoked 100 cigarettes in your life? LS 2. How old were you when you started smoking? LS 3. How many years did you smoke? LS 4. Do you use alcoholic beverages? LS 5. How old were you when you started using alcohol? 0, IA, IB, II, III are degrees of DRS according to the Newton Modified Classification. Being, 0: healthy mucosa; IA: petechiae in normal palatal tissue; IB: localized area of inflammation of the denture-bearing area; II: generalized area of inflammation of the denture-bearing area; III: hyperplasic palatal surface with inflammations of the denture-bearing area.

**Table 3:** Descriptive and inferential analysis of the use of medicaments, salivary flow related to degree of DS, and use of medicaments related to salivary flow

Medicaments’ classification	Total	Number of participants by degree of DS					Correlation to DS		Correlation to SF	
		0	IA	IB	II	III	r	p-value	r	p-value
Do not use medication	34	4	12	9	5	4				
**Antihypertensive (Total)**	107	17	19	33	24	14	-0.029	0.69	0.018	0.8
Diuretics	27	8	4	5	6	4				
Beta blockers	23	5	3	4	6	5				
Angiotensin II Receptor Blocker	63	9	12	22	10	10				
Angiotensin Converting Enzyme Inhibitor (ACE Inhibitor)	24	5	4	7	6	2				
Calcium Channel Blocker	12	3	2	3	2	2				
**Antidiabetics (Total)**	39	11	9	7	8	4	-0.164	0.057	-0.007	0.9
Biguanides/Metformin	28	8	5	4	7	4				
Insulin	12	2	4	2	3	1				
**Antidepressants (Total)**	38	5	4	14	9	6	0.068	0.356	**-0.129**	0.038*
Selective serotonin reuptake inhibitors/Sertraline	14	3	2	3	4	2				
Benzodiazepines/Clonazepam	11	2	2	3	1	3				
Fluoxetine	7	1	0	3	0	3				
Unspecified psychotropic	14	2	0	7	5	0				
**Others (Total)**	106	13	13	40	24	16	0.117	0.079	-0.48	0.443
Hormones	15	2	1	5	5	2				
Proton-pump inhibitors/ Omeprazole	25	4	3	10	4	4				
Anticoagulants	47	6	5	18	10	8				
Statins	42	6	3	15	13	5				
Vasodilator/ Venotonic Drugs	7	4	1	1	1	0				
**Salivary flow rate (SF)**		**Quantitative salivary flow (mL/minute)**					**-0.1934**	0.009*		
Median		0.50^A^	0.40^AB^	0.35^AB^	0.47^AB^	0.35^B^				
Confidence interval		0.5; 0.8	0.4; 0.7	0.4; 0.5	0.4; 0.6	0.3; 0.4				
Maximum; minimum		0.1; 1.6	0.1; 1.4	0.08; 1.2	0.06; 1.5	0.2; 0.9				

Spearman's test (p<0.05)*. DS: Denture Stomatitis. SF: Salivary flow. Equal letters indicate statistical equality. 0, IA, IB, II, III are degrees of DRS according to the Newton Modified Classification. Being, 0: healthy mucosa; IA: petechiae in normal palatal tissue; IB: localized area of inflammation of the denture-bearing area; II: generalized area of inflammation of the denture-bearing area; III: hyperplasic palatal surface with inflammations of the denture-bearing area.

As for oral and denture history, these factors showed that patients that never visited dentists or visited dentists only in emergencies presented a higher degree of DS. Moreover, patients with complete mandibular dentures over six years old or more presented a positive correlation with DS, as shown in Table 4. The *Kapur* index did not correlate significantly with the degree of DS (r=0.114; p=0.07).

**Table 4:** Descriptive and inferential analysis of the correlation of dental and oral history, and dentures of the participants with degree of DS

	Questions	Frequency n (%) of DS' degree					Correlation to DS	
		0	IA	IB	II	III	r	P-value
**Q1.**	Excellent	4 (14.8)	0 (0.0)	1 (1.7)	0 (0.0)	0 (0.0)	0.107	0.091
	Very good	1 (3.7)	3 (9.1)	2 (3.4)	0 (0.0)	0 (0.0)		
	Good	15 (55.6)	15 (45.5)	32 (54.2)	24 (63.2)	15 (57.7)		
	Reasonable	5 (18.5)	13 (39.4)	17 (28.8)	9 (23.7)	7 (26.9)		
	Bad	2 (7.4)	2 (6.1)	7 (11.9)	5 (13.2)	4 (15.4)		
**Q2.**	2x or more/year	0 (0.0)	4 (12.1)	1 (1.7)	1 (2.6)	0 (0.0)	-0.127	0.041*
	1-2x/year	3 (11.1)	2 (6.1)	5 (8.5)	2 (5.3)	3 (11.1)		
	Less 1x/year	2 (7.4)	2 (6.1)	4 (6.8)	5 (13.2)	2 (7.4)		
	Emergency only	16 (59.3)	10 (30.3)	16 (27.1)	12 (31.6)	5 (18.5)		
	Never	6 (22.2)	15 (45.5)	33 (55.9)	18 (47.4)	17 (63.0)		
**Q3.**	Never	25 (96.2)	27 (81.8)	51 (86.4)	33 (86.8)	25 (92.6)	-0.005	0.937
	Rarely	0 (0.0)	2 (6.1)	4 (6.8)	1 (2.6)	1 (3.7)		
	Sometimes	0 (0.0)	3 (9.1)	3 (5.1)	2 (5.3)	1 (3.7)		
	Often	1 (3.8)	1 (3.0)	1 (1.7)	2 (5.3)	0 (0.0)		
**Q4.**	Never	25 (96.2)	30 (90.9)	54 (91.5)	33 (86.8)	26 (96.3)	0.020	0.764
	Rarely	0 (0.0)	1 (3.0)	2 (33.3)	2 (5.3)	1 (3.7)		
	Sometimes	1 (3.8)	1 (3.0)	3 (5.1)	2 (5.3)	0 (0.0)		
	Often	0 (0.0)	1 (3.0)	0 (0.0)	1 (2.6)	0 (0.0)		
**Q5.**	0-5 years	3 (11.5)	0 (0.0)	5 (8.5)	0 (0.0)	1 (3.7)	-0.042	0.510
	6-15 years	4 (15.4)	2 (6.1)	4 (6.8)	8 (21.1)	5 (18.5)		
	16-25 years	7 (26.9)	3 (9.1)	8 (13.6)	6 (15.8)	7 (25.9)		
	Above 25 years	12 (46.2)	28 (84.8)	42 (71.2)	24 (63.2)	14 (51.9)		
**Q6.**	0-5 years	12 (46.2)	14 (42.4)	13 (22.4)	9 (23.7)	10 (37.0)	0.073	0.240
	6-15 years	11 (42.3)	7 (21.2)	24 (41.4)	16 (42.1)	11 (40.7)		
	16-25 years	2 (7.7)	4 (12.1)	8 (13.8)	5 (13.2)	2 (7.4)		
	Above 25 years	1 (3.8)	8 (24.2)	13 (22.4)	8 (21.1)	4 (14.8)		
**Q7.**	0-5 years	14 (63.6)	14 (56.0)	11 (22.9)	9 (31.0)	5 (27.8)	0.182	0.009**
	6-15 years	6 (27.3)	3 (12.0)	20 (41.7)	11 (37.9)	8 (44.4)		
	16-25 years	2 (9.1)	5 (20.0)	8 (16.7)	4 (13.8)	2 (11.1)		
	Above 25 years	0 (0.0)	3 (12.0)	9 (18.8)	5 (17.2)	3 (16.7)		

Kendall's tau_b test (p< 0.05)*. (p<0.001)**. DS: Denture Stomatitis. Questions (Q): Q1- In general, you would say that your oral health is?; Q2- How often do you see a dentist?; Q3- Presence of burning on the palate?; Q4- Presence of burning on the tongue?; Q5- How many years are you edentulous?; Q6- How old is your current maxillary denture?; Q7- How old is your current mandibular denture? 0, IA, IB, II, III are degrees of DRS according to the Newton Modified Classification. Being, 0: healthy mucosa; IA: petechiae in normal palatal tissue; IB: localized area of inflammation of the denture-bearing area; II: generalized area of inflammation of the denture-bearing area; III: hyperplasic palatal surface with inflammations of the denture-bearing area.

For the correlation between the microbial load and the degree of DS (Table 5), the CFU count of the individually identified microorganisms was used. The results showed a positive correlation between the CFU count of *Candida* spp. (denture: r=0.27, p<0.001; palate: r=0.28, p<0.001); *C. albicans* (denture: r=0.22, p=0.004; palate: r=0.22; p=0.003); and *C. glabrata* (denture: r=0.22, p=0.004; palate: r=0.25, p=0.001) with the degree of DS. For the count of enterobacteria (r=-0.24, p=0.002) and *Staphylococcus* spp. (r=0.22; p=0.004) of the palate samples, the results showed negative correlations with the degree of DS. There was no correlation between microbial load and the degree of DS for *C. tropicalis*, other species of *Candida*, and *S. mutans*.

**Table 5:** Descriptive and inferential analyses of the CFU + 1 count (Log 10) of *Candida* spp.; *C. albicans*, *C. tropicalis*; *C. glabrata*, other species of *Candida*, *enterobacteria*, *Staphylococcus* spp., and *Streptococcus mutans* on the denture, and correlation to the degree of DS

CFU count of microbiota			DS' degree					Correlation to DS	
			0	IA	IB	II	III	r	p-value
*Candida spp.*	D	Median	0.0A	3.5AB	4.9B	4.6B	5.0B	0.27	0.000**
		CI	1.0;2.9	2.2;3.8	3.6;4.7	3.1;4.5	3.1;5.0		
	P	Median	0.0A	0.0AB	0.0B	1.6B	0.0AB	0.31	0.000**
		CI	-0.0;0.5	-0.0;0.7	0.7;1.6	1.0;2.0	0.5;1.6		
*C. albicans*	D	Median	0.0A	2.7AB	4.2AB	4.3AB	4.3AB	0.22	0.002*
		CI	0.9;2.6	1.6;3.3	2.5;3.8	2.5;4.1	2.4;4.4		
	P	Median	0.0A	0.0AB	0.0AB	0.0B	0.0AB	0.25	0.001*
		CI	-0.0;0.3	-0.1;0.4	0.3;0.9	0.6;1.5	0.1;0.8		
*C. tropicalis*	D	Median	0.0	0.0	0.0	0.0	0.0	0.5	0.44
		CI	-0.2;0.6	NV	0.1;0.9	0.2;1.6	0.5;2.2		
	P	Median	0.0	0.0	0.0	0.0	0.0	0.10	0.18
		CI	NV	NV	-0.0;0.2	0.1;0.8	0.2;1.2		
*C. glabrata*	D	Median	0.0A	0.0A	0.0AB	0.0AB	0.0AB	0.22	0.004*
		CI	0.0;1.4	0.2;1.1	1.2;2.4	0.5;1.9	0.1;1.7		
	P	Median	0.0A	0.0A	0.0AB	0.0AB	0.0AB	0.27	0.000**
		CI	-0.1;0.3	-0.1;0.4	0.1;0.7	0.0;0.5	-0.1;0.8		
Other species of *Candida*	D	Median	0.0	0.0	0.0	0.0	0.0	0.06	0.37
		CI	-0.0;0.5	-0.0;0.7	0.1;1.0	-0.1;0.4	-0.2;0.9		
	P	Median	2.5	1.6	0.0	0.0	0.0	0.04	0.54
		CI	2.1;2.8	1.0;1.7	NV	-0.1;0.3	NV		
*Enterobacteria*	D	Median	3.8	1.6	2.0	2.3	2.5	0.09	0.20
		CI	3.1;4.2	1.1;2.5	1.5;2.7	2.0;3.5	2.1;4.0		
	P	Median	2.9B	0.0A	0.0A	1.6AB	0.0A	-0.24	0.001*
		CI	2.6;3.2	0.8;1.8	0.7;1.6	1.2;2.1	0.6;1.9		
*Staphylococcus spp.*	D	Median	2.5	1.6	1.8	2.0	1.9	-0.11	0.09
		CI	2.1;2.8	1.0;1.7	1.2,1.8	1.3;2.0	1.1;2.2		
	P	Median	2.4B	1.7B	1.6AB	1.6AB	0.0A	-0.20	0.008*
		CI	1.6;2.4	1.2;2.0	1.1;1.7	1.0;1.9	0.5;1.5		
*S. mutans*	D	Median	1.9	3.3	4.4	3.8	4.2	0.08	0.28
		CI	1.5;4.0	2.5;4.2	3.0;4.5	3.1;4.7	2.1;4.5		
	P	Median	0.0	1.3	1.8	2.4	1.3	0.03	0.69
		CI	0.8;2.1	0.8;1.8	1.2;2.1	1.4;2.4	0.7;1.8		

Spearman's test (p<0.05)* (p<0.001)**. D: denture. P: Palate. CI: Confidence Interval. NV: There was no variation. Equal letters indicate statistical equality. 0, IA, IB, II, III are degrees of DRS according to the Newton Modified Classification. Being, 0: healthy mucosa; IA: petechiae in normal palatal tissue; IB: localized area of inflammation of the denture-bearing area; II: generalized area of inflammation of the denture-bearing area; III: hyperplasic palatal surface with inflammations of the denture-bearing area.

**Figure 2:**
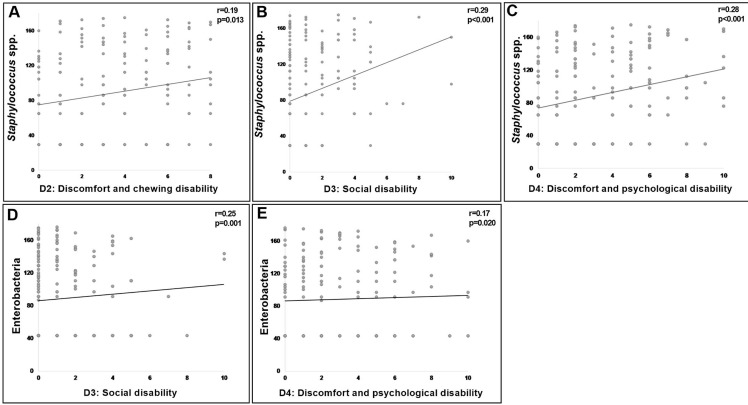
Correlation between OHRQoL, count of *Staphylococcus* spp., and enterobacteria on the mucosal palate

Finally, all the risk factors evaluated, regardless of whether they showed a significant correlation with DS, were submitted to a correlation analysis with the OHRQoL. However, only the CFU count on the palate presented a significant correlation, as shown in Figure 2. *Staphylococcus* spp. may have a low positive correlation with discomfort and chewing disability (domain 2; r=0.19, p=0.013), social disability (domain 3; r=0.3, p<0.001), and discomfort and psychological disability (domain 4; r=0.3, p<0.001). Enterobacteria were related to social disability, and discomfort and psychological disability (domains 3 and 4; respectively; r=0.25, p=0.001; r=0.17, p=0.02) of OHIP-EDENT. It is noteworthy that all factors were evaluated concerning quality of life. Only D1 showed no correlation with any of the variables assessed.

Each point corresponds to the mean rank of correlation between colony-forming units of microorganisms and domains of quality of life.

## Discussion

Female, the onset of alcohol consumption in young adulthood (18–27 years), the presence of *Candida* spp., *Candida albicans*, and *Candida glabrata* on dentures and palate, and salivary flow were associated with denture stomatitis, and the null hypothesis was therefore rejected.

Among the sociodemographic data studied, there was a correlation only between sex and degree of DS. The greater severity of DS was correlated with females. The literature suggests that this relationship is due to attitudinal and physiological issues.^4^

Concerning attitude, women seek dental treatment more frequently than men, in addition to wearing their dentures for prolonged periods.^24^ However, the logic of this explanation is different from the achieved results. Frequent visits to the dentist should reflect better care and guidance about oral health and a lower rate of DS.^24^ Moreover, another fact that could be considered is that the sample was predominantly made up of women, which could lead to the hypothesis of a greater number of reported cases of denture stomatitis, opposite to what was observed in males, which could represent a situation of underreported cases of denture stomatitis. However, this epidemiological profile and its correlation with DS could be addressed in future studies to confirm or refute such hypotheses.

Regarding physiological issues, hormonal changes before and after menopause contribute to the atrophy of bone support, which causes chronic irritation in poorly adapted dentures.^4,25^ The women in this study were aged over 59 years; therefore, they were all post-menopausal, corroborating this hypothesis in the literature. It was expected that age, education level, and income would correlate with DS, but this was not observed in the results. Although correlated with DS, the information on marital status and “lives with” was intended to characterize the sample, and no significant correlation results were expected.

Regarding lifestyle, there was an unexpected correlation between never consuming alcoholic beverages and a higher degree of denture stomatitis. Many patients (n=97, 52.7%) said they had never consumed alcohol. Although the number of patients is considerable, this relationship should be interpreted cautiously, since alcohol can be related to lesions in epithelial tissue.^26^ However, when assessing the patients who reported consuming alcoholic beverages (n=85, 46.2%), it was noted that the onset of alcohol consumption in young adulthood (18–27 years) was a determining factor for the positive correlation with denture stomatitis. Although alcohol metabolism occurs predominantly in the liver, this process also appears in the oral mucosa due to dehydrogenase and microorganisms capable of breaking down alcohol into acetaldehyde.^26^ Acetaldehyde is a mutagenic and carcinogenic substance that can cause significant harm. The effects of acetaldehyde on human cells include interfering with the synthesis and reparation of DNA; inducing exchanges between sister chromatids; producing gene mutations; inhibiting the enzyme O6-methylguanitransferase (responsible for repairing injuries to DNA caused by alkylating agents); and binding cellular proteins and DNA, resulting in morphological and cellular damage. Thus, long-term alcohol exposure (12 months) can result in dysplastic changes with keratosis, reducing epithelial thickness, which contributes to the appearance of inflammatory reactions.^26^

Another possible risk factor to be considered is systemic causes related to hyposalivation, which may be due to local cancer therapy, psychological disorders, autoimmune diseases, and certain medication, such as antidepressants, antihypertensives, and diuretics.^27^ The use of medication is one of the leading causes of xerostomia, but it is rarely associated with irreversible diseases of the salivary glands. Regarding medication use, only 34 (18.5%) of the participants reported not using any medication. The most used medications were antihypertensives (n=107, 58.2%), antidiabetics (n=39, 21.2%), and antidepressants (n=38, 20.7%). These examples of medical history and medication use represent a scenario of elderly patients commonly reported in other studies.^27,28^

The patients who used a class of antidepressants showed a negative correlation with salivary flow, and this factor had a negative correlation with DS severity, different to what was reported by Lucena, et al.^27^(2010).

It was observed that patients who went to the dentist only for emergencies or who never went had a positive correlation with a higher degree of DS. The patient’s lack of visits to the dentist contributes to a lack of information regarding hygiene methods, which contributes to the severity of stomatitis. Also, in most patients DS can manifest asymptomatically, adding to the impossibility of self-diagnosis, and can establish itself for long periods without any diagnosis. In previous studies, a relationship between the frequency of visits to the dentist and DS was determined.^29^

Regarding denture history, it was observed that patients who had complete mandibular denture for over six years or more presented a positive correlation with DS. It was expected that this correlation would be with the maxillary denture, the region with the highest prevalence of mucosal lesions,^7^ nonetheless, one possibility to justify the longer use of the mandibular denture and the higher degree of DS is a possible change in the vertical dimension of occlusion, an important factor that correlates with the trauma factor of DS. However, this study did not directly evaluate the vertical dimension of occlusion, but in the literature it is established that this factor may have a direct relationship with mucosal lesions in complete edentulous patients.^16^

Concerning anatomical features and their correlation with DS, to our knowledge, this is the first article to report these factors. Although the results indicate no significant correlation between the factors, it is believed that different residual ridges, muscle insertions, and mucosal resilience are relevant clinical information for a favorable or unfavorable prognosis regarding the quality of the dentures,^21^ which is directly related to the etiologies of mucosal epithelial lesions.

The sample for this study was composed of participants from the public health system in the city of Ribeirão Preto and the surrounding region, who sought care at the School of Dentistry of Ribeirão Preto for replacement dentures. They showed characteristics common to the South American edentulous population,^4,13,19,20^ and the results showed that the main factor involved in the risks related to DS was the presence of biofilm rich in pathogenic microorganisms, possibly due to a failure of maintaining oral health and dentures.

The microorganisms *Candida* spp., enterobacteria, *Staphylococcus* spp., and *S. mutans* were selected and evaluated because they are species commonly found in the biofilm of complete dentures,^13,20^ *Candida albicans* in particular being highly correlated with the presence of DS.^20,30^ In this study, positive correlations were found between CFU of *Candida* spp., *C. albicans*, and *C. glabrata* (dentures and palate) and a higher degree of DS. The highly effective adhesion of these microorganisms to the denture acrylic base justifies this finding, which corroborates several studies already reported in the literature.^4,20,25^ This result confirms the need for special care with denture hygiene as a form of preventing DS,^31^ since hygiene contributes to a reduction in biofilm and microbial load, promoting better contact between the inner surface of the dentures and the mucosa, which decreases the risk of worsening systemic conditions due to the opportunism of the microorganism.^8,16,17,20^ Therefore, well-established management incorporated into daily practices related to oral and denture hygiene may be essential to control and prevent DS. In this scenario, future controlled clinical studies could compare individuals before and after DS treatment, including in other populations such as younger patients, to highlight the relationship between microbial load and DS.

When studies specifically evaluated the biofilm of gram-positive bacteria, such as *Staphylococcus* spp., it was observed that their adherence to surfaces exhibited different behaviors compared to *C. albicans*. Regarding biofilm formation, studies have shown that *C. albicans* can form a more structured and complex biofilm when compared with *S. aureus*, presenting better adherence and a more homogeneous biofilm-like structure, which seems to adhere mainly as individual or grouped cocci, and biofilm-like structures attached to the different morphological forms of the fungus.^32^ The microbial count of *Staphylococcus* spp. had a negative correlation with the degree of DS, which can be explained by the lower adhesion of these microorganisms to the surfaces evaluated.^32^

A recent literature review^33^ showed that when the two microorganisms are together, *S. aureus* and *C. albicans* coinfection alters the microbial metabolism related to infection with either organism alone. Metabolic changes during coinfection regulate virulence, such as increased toxin production in *S. aureus* or contribution to morphogenesis and cell wall remodeling in *C. albicans*. Both also form polymicrobial biofilms, which have greater biomass and lower susceptibility to antimicrobials relative to mono-microbial biofilms. The *S. aureus* and *C. albicans* metabolic programs induced during coinfection have an impact on interactions with host immune cells, resulting in more pronounced microbial survival and immune evasion.^33^ However, in this study, the coinfection of *Staphylococcus* spp. with *C. albicans* was not evaluated, but it is clear that the microbial load of *Candida* spp. and *C. albicans* was significant and may possibly have been influenced by this coinfection, which would be an interesting assessment to address in future studies, particularly in patients who do not respond to appropriate initial therapy.^33^

For *S. mutans*, no correlation with DS was found. This may be due to the fact that it is a microorganism related to the initial adhesion of microorganisms, being a precursor to biofilm formation.^12,20^ As most of the patients evaluated had been using their dentures for a long time, it is believed that the dentures deposits already presented a more complex structure of the biofilm collected.

Although the literature suggests that chronic DS-type injuries can influence the quality of life, there is still no direct evidence of this relationship when considering denture stomatitis.^24^ According to the authors of this study, this is the second time that this relationship has been reported.^19,24^ However, for microbial load, which is one of the factors strongly related to DS, there is still no data on its relationship with quality of life.

Although *Staphylococcus* spp. showed a negative correlation with the degree of DS, the patients with a higher CFU of *Staphylococcus* spp. had a lower quality of life. It was expected that the microbial load of other microorganisms, especially *Candida* spp., would also correlate with the quality of life. However, it can be assumed that *Staphylococcus* spp. can coexist with other microorganisms,^33^ potentiating severity and symptomatology, mainly on the palate, the region with the greatest manifestation of DS.^7^ However, the microorganisms were evaluated separately in relation to quality of life.

*Staphylococcus* spp. showed a positive correlation with poor quality of life in three domains (D2: Psychological discomfort and disability; D3: Social disability; and D4: Oral pain and discomfort), enterobacteria with two domains (D3 and D4), and for the other microorganisms no significant correlation was observed between CFU and quality of life. This may be due to the fact that local chronic inflammation, mediated by microorganisms, is related to epithelial lesions that can trigger edematous ulcerated sites, with symptoms of pain and burning, commonly seen in DS,^7^ which can interfere with the intimate adaptation of the dentures to the supporting tissue, and impact on patient satisfaction and quality of life.^19^ Additionally, the hypothesis that microorganism presence could promote injured tissue and, consequently, pain and discomfort, can be reinforced by the correlation between microbial load and quality of life.^19^

A further problem was the domain of social disability, that is, the impact of oral conditions on social relationships. Those who experienced embarrassing situations and less tolerance from their spouse and family had a higher CFU of *Staphylococcus* spp. and enterobacteria. This result may be related to the microorganisms identified in the denture and palate, which promoted staining and odors, signs and symptoms that can cause discomfort and insecurity in these participants’ social life.^19,24^

This study presented some limitations. One of these was not evaluating the quantity of biofilm on the two surfaces (denture and palate). However, the presence of a microbial load of all pathogens may suggest that biofilm was present on these surfaces. Another area of limitation is the design of a cross-sectional study without the power of a justified causal relationship between all the factors of DS and quality of life. Future research should clarify this relationship using a randomized and controlled study to clarify cause–effect relations.^16^

Other limitations of this study are that the denture vertical occlusal dimension, retention, and stability were not evaluated to quantify them as possible risk factors. Only one clinical evaluation was carried out and did not include individuals with evident denture quality problems. However, these are necessary factors to consider in future studies.

Finally, one significant limitation is that the sample size was an estimate based only on the colony-forming unit (CFU) counts, information commonly presented in previous studies. However, many risk factors for DS should be investigated by controlled studies. To this end, the results of this study can contribute to future sample estimates.

## Conclusion

Based on the findings of this clinical study, the main risk factors for developing denture stomatitis are:

1. Being female, consuming alcoholic beverages as a young adult (18–27 years), and the presence of *Candida* spp., *Candida albicans*, and *Candida glabrata* on the dentures and palate.
2. The quality of life, especially domains of OHIP-EDENT D2, D3, and D4 may be slightly influenced by the CFU count of *Staphylococcus* spp., and domains D3 and D4 by enterobacteria. This relationship appears to be potentially significant and should be investigated as a trend in randomized clinical trials.
